# Reimagining Literature Evaluation Pedagogy Through AI-Generated Podcasts: Pharmacy Students’ Perspectives and Critical Appraisal

**DOI:** 10.3390/pharmacy14070145

**Published:** 2026-10-01

**Authors:** Bethany R. Shoulders, Madelyn Hickey, Tracy Leonard, Phuoc (Tyler) Bui, Skylar Johnson, Anthony M. Casapao, Priti Patel, Kaitlin M. Alexander

**Affiliations:** 1 Department of Pharmacy Education and Practice, University of Florida College of Pharmacy, 1225 Center Drive, Gainesville, FL 32610, USAkaitlin.alexander@cop.ufl.edu (K.M.A.); 2 Department of Pharmacy Services, UF Health Shands Hospital, 1600 SW Archer Rd., Gainesville, FL 32608, USA; 3 Department of Curricular Affairs and Accreditation, University of Florida College of Pharmacy, 1225 Center Drive, Gainesville, FL 32610, USA

**Keywords:** podcast, critical appraisal, student perspective, artificial intelligence

## Abstract

This study explores pharmacy students’ perspectives on applying their newly formed literature evaluation skills to evaluate AI-generated podcasts. The podcasts were short overviews of the articles assigned to the students to present to facilitators. Academic staff used each study article to generate podcasts with an AI platform. Students listened to an AI-generated podcast on their assigned article following a skills assessment and provided an appraisal of the information. Academic staff prompted them to evaluate the podcast for accuracy, misleading and omitted information, and their desire to utilize an AI podcast for their learning in the future. Qualitative evaluation of the students’ responses was performed using coding of “agreement, disagreement, or neutral” by three academics. If a student did not address a prompt on the discussion board, other components of their response were still considered. In total, 230 responses were evaluated, and most students agreed that the podcast was accurate (169/230, 73%), not misleading (125/217, 58%), but neglected to include important information (168/218, 77%). Most students (124/227, 55%) agreed that they would use the AI-generated tool for literature evaluation in the future; however, students who disagreed with future use cited its oversimplification and generality. This educational activity required students to engage with an AI-generated podcast after completing a literature evaluation and to evaluate its effectiveness as an adjunctive learning modality.

## 1. Introduction

Artificial intelligence (AI) is transforming many different professional practices, including pharmacy. Within pharmacy practice, AI is used to identify adverse drug events and drug interactions, support clinical decision making, and evaluate the primary literature [1]. The rapid integration has led to the World Health Organization (WHO) publishing guidance to support the development of policies and practices for incorporating AI into healthcare, including healthcare education [2]. The organization emphasizes that the evolving capabilities of large language models (LLMs) will require healthcare professionals to engage in ongoing training and adaptation. Consequently, pharmacy educators must identify effective strategies to ensure that students are prepared for the integration of AI into clinical practice, including recognizing its limitations.

While AI has the potential to enhance the training of healthcare professionals, it also poses risks, including the possibility that students may rely too heavily on AI and forgo the learning process [2]. Academics must manage several risks when incorporating AI into teaching: the loss of previously learned skills (deskilling), the development of faulty reasoning from over-reliance on AI (mis-skilling), and the absence of foundational skills being developed at all (never-skilling) [3]. The use of AI in pharmacy education has been met with mixed reactions by both educators and students that identify the value and limitations of AI as a learning tool [1,4]. Therefore, strategic and creative approaches to incorporating AI into pharmacy skills, such as literature evaluation, are needed.

AI has the capacity to facilitate primary literature overviews and serve as a useful resource for clinicians and students; however, its known limitations include overlooking key references and the lack of nuanced interpretation and critical analysis of scientific articles [5]. A novel way to expose students to AI is by having them engage with podcasts to support learning [6]. Podcasts provide a flexible and engaging educational method, particularly for auditory learners, and facilitate self-paced learning that can be an adjunct or supplement to traditional lectures [7]. Several studies have shown that healthcare students, including pharmacy students, use or support the use of podcasts as supplemental learning tools [6,8,9,10]. Furthermore, these tools are used by healthcare professionals in practice to facilitate continuing education [11,12,13]; therefore, understanding the role of podcasts as a supplemental learning tool is important.

Generative AI has expanded the ability to create customized educational materials, including podcasts [14]. However, evidence on the implementation of AI-generated podcasts as a teaching tool in pharmacy skills education, such as literature evaluation, is limited [1,10]. The previous literature has focused on pharmacy students’ utilization of AI-generated podcasts as an innovative instructional strategy or on educators’ evaluations of their accuracy but has not examined students’ appraisal of podcast content [10,15]. This study aimed to evaluate students’ perceptions of an AI-generated podcast’s ability to discuss a primary literature article and their willingness to use AI-generated podcasts for future learning. The primary focus of the activity was to engage students in applying their literature evaluation skills to evaluate AI-generated content.

## 2. Materials and Methods

### 2.1. Study Purpose

In this study, academic staff generated podcasts using an AI tool for students to listen to after they presented their first literature evaluation in the Doctor of Pharmacy curriculum. Students listened to the podcast and then completed an appraisal of the content. The purpose of this learning activity was to give pharmacy students an opportunity to interact with the AI-generated content and apply their literature evaluation skills in this critical appraisal.

### 2.2. Study Context

This study was conducted at an institution that has embraced using AI in teaching and learning as well as exposing learners to its use in practice; students have access to many AI tools/resources that are data-protected by the university. Our students have reported general knowledge and experience with generational AI tools [4]. Furthermore, this specific cohort of students received instructions in their first year of the Doctor of Pharmacy curriculum at the University of Florida College of Pharmacy (USA) on how to use the AI tools provided by our institution. This educational activity was implemented in August 2024 during the second year of the Doctor of Pharmacy curriculum in a Pharmacy Skills course. Literature evaluation skills are introduced in the first year of the curriculum, but a comprehensive assessment that includes biostatistics, literature appraisal, and a presentation in the form of a “journal club” is not carried out until this Pharmacy Skills course. The Pharmacy Skills course is considered a “laboratory” course where skills are practiced and evaluated in small groups and individually. “Journal clubs” are performed twice every academic year in this course. The first time this skill is evaluated, students are given questions to prompt their literature evaluation, and the second time, they are not given questions to prompt their review. In both iterations, students are assigned to read three primary literature articles and present one article, prepared in pairs or groups of three per article, to their team of five to six students. The AI-generated podcasts were introduced after the first session evaluating this skill.

### 2.3. Educational Activity Design

The educational activity included flipped classroom lectures on “Preparing for Journal Club” and a “Journal Club Statistics Review.” Students were expected to read three journal articles and were assigned one of the three articles to present answers to guided questions in pairs or groups of three. Group of less than six were assigned two articles to present and were instructed to be prepared to discuss the third article with their facilitator. The three articles that were assigned to students were randomized controlled trials. The topics included a comparison of three antibiotics used for urinary tract infections (UTIs) caused by extended-spectrum beta-lactamase (ESBL)-producing organisms, a food supplement’s impact on the prevention of recurrent UTIs, and a non-inferiority trial comparing non-steroidal anti-inflammatory drugs (NSAIDs) to antibiotics in the treatment of uncomplicated UTIs [16,17,18]. These articles were chosen for their similar structure, and they incorporated clinical content that students were familiar with. Before the skill evaluation, students were also expected to access a patient case via a mock electronic health record and decide if they could apply their article to the treatment of that patient.

In the classroom, students presented their findings from their assigned article and were evaluated by facilitators on their participation, quality of comments, and listening skills. Facilitators ensured that their presentations were accurate and reviewed every article with each group. For groups of less than six, the remaining article was thoroughly discussed within the small group. Once their presentations were completed, students listened to podcasts on their assigned articles generated by the course academics from NotebookLM 2.0 (Google (Google Labs), Mountain View, CA, USA) [14]. Academic staff generated these podcasts using the articles as source materials and selected a short “audio overview.” They were all generated the same day to ensure the use of the same AI technology, and no additional prompts were given to the AI to influence the content of the podcast. Staff provided a review of the content before it was uploaded to the learning management system for students to review. Students were instructed to take notes on key points, insights, and any questions that arose and pay attention to how the podcast presented the information, the depth of content, and any unique perspectives offered. After listening, they were assigned questions to consider and instructed to post their comments on the course discussion board. Posts were mandatory and were assigned a grade of completion but did not count towards the final grade in the course. This critical appraisal prompted students to identify the article reviewed and share their perspectives on whether the podcast was accurate, misleading, or neglected to include any important points. Finally, students were asked if they would create and utilize an AI podcast in the future Details on these instructions are found in Table 1, and the content and timeline of this educational activity are displayed in Figure 1.

### 2.4. Study Outcomes and Statistical Analysis

Student discussion board posts were evaluated qualitatively by using a directed content analysis. Comments from students were organized by article type and then grouped by topic/question. Three academic staff coded responses to accuracy, misleading information, and neglected information as “yes, no, and neutral” and responses to utilization in the future as “yes, no, and maybe.” If a student did not address a question, other components of their response were still considered. Academics initially coded the responses independently. They then met to discuss any discrepancies and reached a verbal consensus.

Descriptive statistics were performed on the coded responses, and responses were stratified for each article. Specific responses for use in the future were reviewed to gain insight into reasons for adoption or hesitation to use this tool. Comparisons between articles were performed via Chi-squared test for independence or Fisher’s exact test. Statistics were performed using JMP Student Edition, version 19 (JMP Statistical Discovery LLC, Cary, NC, USA) [19]. This study was approved by the University of Florida Human Research Protection Program as exempt (Protocol Number: ET00055529).

## 3. Results

Two hundred thirty responses (n = 230) were evaluated, which represented all students present for the class; 85 were assigned the article on antibiotics for ESBL UTIs, 95 commented on the food supplement to prevent recurrent UTIs article, and 50 students responded to the podcast on the NSAIDs vs. antibiotics for uncomplicated UTIs article. All described the accuracy of the podcast in their responses, but 13 students did not comment on misleading information, and 12 did not discuss omitted information. Most students agreed that the podcast was accurate (169/230, 73%), not misleading (125/217, 58%), but neglected to include important information (168/218, 77%). The students rarely thought the podcast was not accurate (9/230, 4%), but more thought it was misleading (70/217, 32%). These responses differed somewhat when stratifying by article type. Numerically, there were more students who reviewed the podcast for the article on the food supplement for the prevention of UTIs who believed it was accurate and less students that were neutral on this question (15% vs. 28% in other groups, *p* = 0.053). Students also perceived that the podcast generated from this article was less misleading than others. Twenty percent answered “yes” to misleading on the food supplement podcast, while 37% answered “yes” on the antibiotics for ESBL UTIs podcast, and 48% answered “yes” on the NSAIDs vs. antibiotics for uncomplicated UTIs podcast (*p* = 0.003). Detailed information on the responses stratified by the three articles is included in Table 2.

When responding to creating an AI podcast in the future to support preparation for literature evaluation, most students answered “yes” (124/227, 55%), while 20% gave responses equivalent to “maybe” (45/227), and 25% provided a “no” response (58/227). Three students did not provide a response to this question. When stratifying responses by the journal article the podcast was generated from, there was a numerical increase in “no” responses from students that listened to the podcast generated from the article on NSAIDs vs. antibiotics for uncomplicated UTIs (38% vs. 20% and 24%, *p* = 0.064). The authors also reviewed the explanations behind these responses to further understand students’ perspectives. Many of the “yes” responses mentioned using it as an engaging supplement to prepare for journal club. “Maybe” responses considered that there may be inconsistencies but would likely make the article easier to digest. On the other hand, “no” responses felt that it would confuse their understanding or oversimplify the findings. Excerpts from these responses are included in Table 3 to provide more context into why the “yes,” “no,” and “maybe” responses were coded as such.

## 4. Discussion

This article presents an innovative strategy for using AI-generated podcasts as a supplemental tool in reinforcing literature evaluation skills. The appraisal of an AI-generated podcast after students were evaluated on a “journal club” presentation offered an additional opportunity for students to evaluate an AI-generated overview of the content. The findings suggest that that although students may be willing to use AI-generated podcasts in the future, applying their literature evaluation skills to critically appraise the podcast enabled them to identify areas for improvement and develop an awareness of the limitations of AI-generated educational content.

In the previous literature, podcasts have been established as a supplement that can promote learning and reflection; however, the evidence behind using AI-generated podcasts is limited [6,8,9]. Podcasts generated by AI using similar methods to our study have been evaluated by academics teaching in health professional education and deemed comparable to human-generated podcasts, but students’ perspectives are missing from this evaluation [15]. Another study that evaluated AI-generated podcast use as a supplement to journal club preparations in pharmacy learners reported 60% of students completely listening to the podcasts, while we were able to achieve 100% participation in our strategic instructional design [10]. While this previous study was designed to understand the uptake of an AI-generated podcast, our findings describe students’ perspectives on the podcasts’ content and assess their potential to utilize it as a supplement in the future. The podcast content was reviewed by pharmacy staff prior to being shared with students; however, the purpose of this evaluation was not to compare students’ critiques with educators’ assessments. Rather, it was designed to provide students with an additional opportunity to apply their content evaluation skills using a different learning modality and to explore their perspectives on appraising AI-generated podcast content. Future studies could examine the accuracy and quality of students’ evaluations of AI-generated podcast content, as well as the impact of AI-generated podcasts on learning outcomes and skill development.

Academic staff strategically introduced the podcast after practicing literature evaluation skills to avoid negatively impacting the learning process. The AI podcast was available only after students had completed an independent assessment of the article and received feedback on their journal-club presentation from session facilitators. Therefore, the assignment of students listening to an AI-generated podcast about an article and providing a critical appraisal on its content served as a supplemental activity to reinforce literature evaluation skills in an engaging format. Furthermore, the majority of students (124/227, 55%) planned to use these podcasts as an overview in the future when preparing for literature evaluation. Beyond reinforcing skills, the activity promoted AI literacy. Students critically assessed the podcast, thereby encountering both the advantages and limitations of using AI for this task. Critical appraisal responses confirmed that the exercise made students aware of AI’s benefits and drawbacks in literature evaluation. Reviewing these responses, academic staff believe that this activity provided value and plan to evaluate the impact on learning outcomes in the future.

Responses varied by the article assigned. In particular, the article on the use of a food supplement to prevent UTIs was less likely to be viewed as inaccurate or misleading. On the other hand, students listening to the podcast generated from the article on NSAIDs vs. antibiotics for uncomplicated UTIs were less likely to use AI-generated podcasts to help prepare for journal club in the future. These discrepancies are likely introduced through the use of three different articles in this cohort. Students could have also been biased in their responses from their knowledge of appropriate treatment of UTIs. Therefore, the statistical comparisons should be viewed as exploratory, and the authors cannot define the exact reason for the variance. While the use of different articles represents a limitation in the evaluation of the response comparisons, multiple articles allow for a more robust discussion and exposure for students in the skill-developing portion of the learning activity. For instance, discussions on non-inferiority statistics and differences in group stratification and blinding practices were able to occur in their small groups. Practically, academic staff chose to use three articles to limit partners within their small groups to two or three students. In the instance that small groups had less than six students, the article on NSAIDs vs. antibiotics for uncomplicated UTIs was presented by the facilitator, and students were not assigned to review this podcast. Therefore, the number of students providing a critical appraisal of this podcast is notably lower (50 vs. 95 and 85 for the other articles). Academic staff considered having all students listen to all podcasts and respond to each; however, specified class time did not allow for this method of teaching. Furthermore, listening to and responding to the podcast generated from their assigned article promoted thoughtful engagement with the literature and facilitated the application of critical appraisal skills in a novel context.

Regardless of the article type, there was a high percentage (77%) of students that felt the podcasts neglected to include information. This finding may be related to the duration of the podcast or the current technology available to create the podcast. However, all podcasts were generated on the same day with the same AI technology and methodology. In the future, academic staff could include more information in a prompt on what content to include to improve the reliability of the podcast. Furthermore, future evaluations could compare educators’ appraisal of the content and compare this to learners’ evaluations. In this instance, the imperfection in the podcasts represents how students may interact with AI in the future and highlights that these podcasts are not designed to be the sole source of information and overview for an article. This realization contributes to learners’ understanding of the appropriate use of AI in clinical settings and suggests that the activity helped increase awareness of AI’s limitations. The belief that the podcasts omitted information might have also impacted their response to the question on utilization in the future.

Limitations to the evaluation of the responses are that students are novices and therefore may not be fully capable of identifying inaccurate, misleading, or omitted information from the podcast. However, they did perform the skill independently and receive feedback on critical literature appraisal from a pharmacist prior to listening and reviewing the podcast content, so their personal critical appraisal should be better informed. However, responses were not evaluated and graded for accuracy. Furthermore, the prompt questions may have introduced bias instead of having an open-ended response; however, the questions were used to standardize the students’ responses and gain insight to their perspectives. Finally, specific information on listening was not captured, and not all students answered each question. Participation in this activity did not necessarily indicate active listening. Future studies should be designed to capture the effect of AI-generated podcasts on learning outcomes, as our study was not designed to evaluate skills performance, and the full impact on learning is unclear.

## 5. Conclusions

This educational activity was intentionally designed to extend students’ literature evaluation skills into a novel context by incorporating the critical appraisal of an AI-generated podcast after completing a traditional literature evaluation. Through this process, students applied and reinforced their evaluation skills while gaining experience with both the potential benefits and limitations of AI-generated educational content. The findings provide insight into students’ perspectives on using AI-generated podcasts in literature evaluation and highlight the value of requiring learners to critically assess, rather than passively accept, AI-generated information. This approach may help promote skill development while increasing awareness of the challenges associated with AI use in educational and clinical contexts. Future research should evaluate the quality and accuracy of students’ critical appraisals and examine the impact of AI-generated podcasts on learning outcomes and literature evaluation skill development.

## Figures and Tables

**Figure 1:**
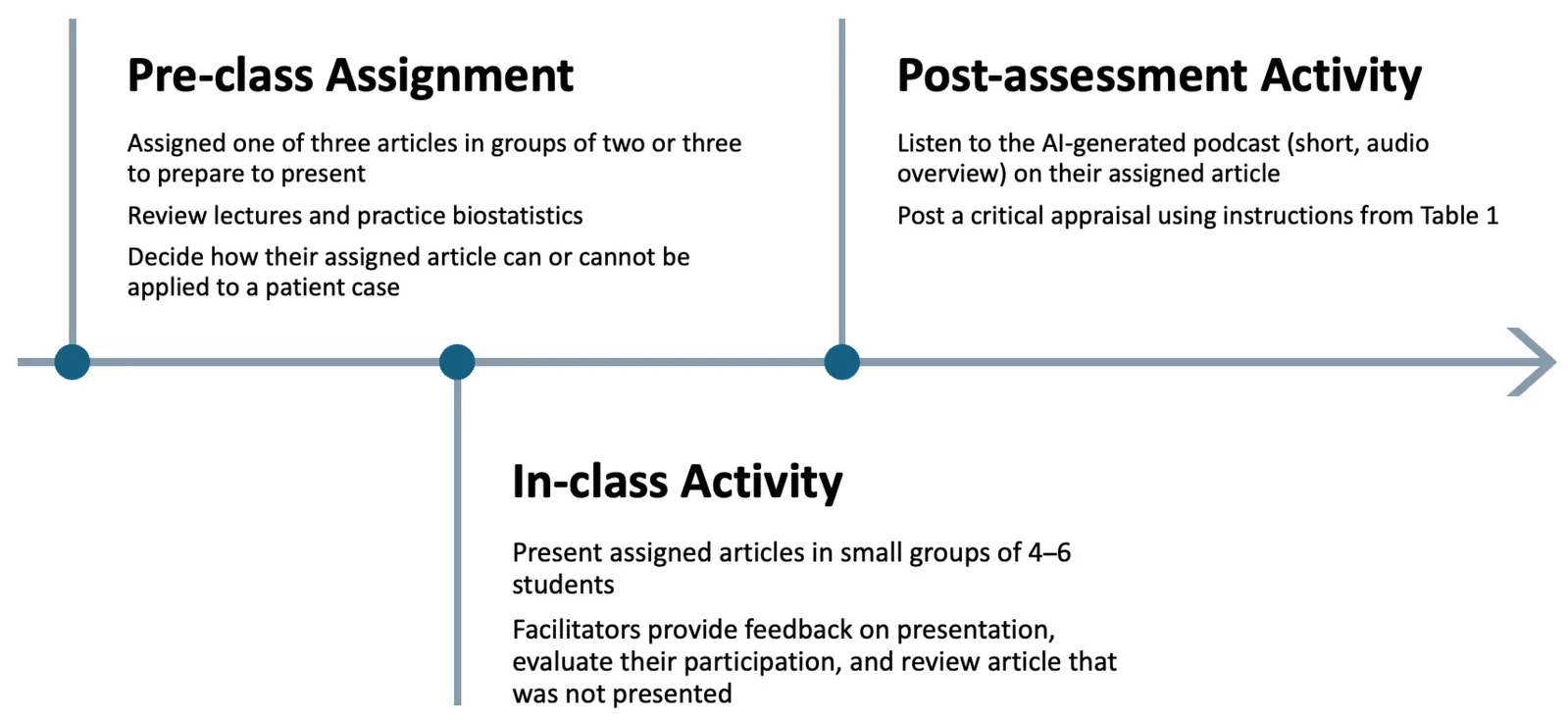
Design of the literature evaluation educational activity.

**Table 1:** Instructions for student critical appraisal on AI podcasts.

Listen to the podcast on your assigned article:
Take notes on key points, insights, and any questions that arise.
Pay attention to how the podcast presents the information, the depth of content, and any unique perspectives offered.
2. Post on discussion board. Posts should include the following responses:
Which article are you discussing?
Was the podcast accurate in describing your article’s methods, results, or conclusions?
Was the podcast misleading in any way?
Did the podcast neglect to include any important points?
Would you create an AI podcast to help you prepare for journal club?

**Table 2:** Student responses stratified by article assignment.

**Podcast Content**	**Yes**	**No**	**Neutral**	**Statistics**
Accuracy, n (%) *				χ^2^ (4, n = 230) = 6.28, *p* < 0.001, Cramér’s V = 0.125
Antibiotics for ESBL UTIs	59 (70)	2 (2)	24 (28)	
Food Supplement for Prevention	76 (80)	5 (5)	14 (15)	
NSAIDs vs. Antibiotics for Uncomplicated UTIs	34 (68)	2 (4)	14 (28)	
Misleading, n (%)				χ^2^ (4, n = 217) = 17.3, *p* = 0.002, Cramér’s V = 0.19
Antibiotics for ESBL UTIs	30 (37)	46 (56)	6 (7)	
Food Supplement for Prevention	18 (20)	56 (63)	15 (17)	
NSAIDs vs. Antibiotics for Uncomplicated UTIs	22 (48)	23 (50)	1 (2)	
Neglected Information, n (%)				χ^2^ (4, n = 218) = 3.46, *p* = 0.483, Cramér’s V = 0.09
Antibiotics for ESBL UTIs	64 (79)	12 (15)	5 (6)	
Food Supplement for Prevention	65 (71)	19 (21)	7 (8)	
NSAIDs vs. Antibiotics for Uncomplicated UTIs	39 (85)	5 (11)	2 (4)	
**Podcast Usefulness**	**Yes**	**No**	**Maybe**	
Use in the Future, n (%)				χ^2^ (4, n = 227) = 5.37, *p* = 0.251, Cramér’s V = 0.11
Antibiotics for ESBL UTIs	50 (60)	17 (20)	17 (20)	
Food Supplement for Prevention	52 (56)	22 (24)	19 (20)	
NSAIDs vs. Antibiotics for Uncomplicated UTIs	22 (44)	19 (38)	9 (18)	

* Fisher’s exact test was performed to generate a *p*-value in this comparison, since 20% of the cells had counts <5.

**Table 3:** Examples of explanations for future use of AI responses.

“Yes” Responses		
“help me grasp the bigger picture and feel more confident”	“makes complex research more accessible and engaging”	“use as a supplement to quickly learn the key points of a study”
“No” Responses		
“confuses my understanding of the article”	“too general to be very helpful”	“oversimplify or misrepresent findings”
“Maybe” Responses		
“there could be inconsistencies”	“would consider to make the article easier to digest”	“way to orient thinking when critically assessing the article”

## Data Availability

The datasets presented in this article are not readily available due to privacy restrictions. Requests to access the datasets should be directed to the corresponding author.
